# Pyrrolizidine Alkaloids and Fatty Acids from the Endemic Plant Species *Rindera* *umbellata* and the Effect of Lindelofine-*N*-oxide on Tubulin Polymerization

**DOI:** 10.3390/molecules180910694

**Published:** 2013-09-03

**Authors:** Boris M. Mandić, Milena R. Simić, Ivan M. Vučković, Ljubodrag V. Vujisić, Miroslav M. Novaković, Snežana S. Trifunović, Snežana D. Nikolić-Mandić, Vele V. Tešević, Vlatka V. Vajs, Slobodan M. Milosavljević

**Affiliations:** 1 Faculty of Chemistry University of Belgrade, Studentski trg 16, Belgrade 11158, Serbia; E-Mails: ivuckovic@chem.bg.ac.rs (I.M.V.); ljubaw@chem.bg.ac.rs (L.V.V.); snezanat@chem.bg.ac.rs (S.S.T.); snezananm@chem.bg.ac.rs (S.D.N.-M.); vtesevic@chem.bg.ac.rs (V.V.T.); smilo@chem.bg.ac.rs (S.M.M.); 2 Faculty of Pharmacy University of Belgrade, Vojvode Stepe 450, Belgrade 11000, Serbia; E-Mail: milena@pharmacy.bg.ac.rs; 3 Institute of Chemistry, Technology and Metallurgy University of Belgrade, Njegoseva 12, Belgrade 11000, Serbia; E-Mails: mironov@chem.bg.ac.rs (M.M.N.); vajs@chem.bg.ac.rs (V.V.V.)

**Keywords:** *Rindera umbellata*, pyrrolizidine alkaloids, fatty acids, tubulin polymerization

## Abstract

The examination of the aerial parts, roots, and seeds of the endemic plant *Rindera* *umbellata* is reported in this paper for the first time. Phytochemical investigation of *R. umbellata* led to the isolation and characterization of ten pyrrolizidine alkaloids and eleven fatty acids in the form of triglycerides. Pyrrolizidine alkaloids **1**–**9** were found in the aerial parts, **7** and **8** in the roots, and **6**–**10**, together with eleven fatty acids, in the seeds of this plant species. The structures of compounds **1**–**10** were established based on spectroscopic studies (^1^H- and ^13^C-NMR, 2D NMR, IR and CI-MS). After trans-esterification, methyl esters of the fatty acids were analyzed using GC-MS. The effect of lindelofine-*N*-oxide (**7**) on tubulin polymerization was determined.

## 1. Introduction

The toxic pyrrolizidine alkaloids (PAs) are a large group of secondary metabolites, and it has been estimated that PA-producing plants represent 3% of all flowering species [1,2,3,4,5,6]. Despite the fact that PAs have been the subject of investigations for many years, interest in them remains. The investigations of the activities of plant PAs have indicated their neurotoxic, mutagenic, carcinogenic, but also antitumor effects [7,8,9]. PAs are readily absorbed from the digestive tract and cause harmful effects only after undergoing activation to toxic metabolites in the liver. The effects include a variety of changes in biomolecules leading to permanent damage to genes and chromosomes, the ability of cells to divide, the development of cancer and even cell death. Some PAs are strong toxins for humans and domestic animals [10]. The acute toxicity of PAs varies widely. The rat *LD*_50_ of most alkaloids known to be significant for human health are in the range of 34–300 mg/kg, although some approach 1,000 mg/kg. On the other hand, many species rich with PAs are used in traditional medicine in Asia and Africa, which makes them a very interesting for phytochemical investigation.

The occurrence of PAs in plants is scattered in several unrelated botanic families: Asteraceae, Boraginaceae, Fabaceae, Apiaceae, Convolvulaceae, Celestraceae, Proteaceae, Santalaceae, Sapotaceae, Ranunculaceae, Euphorbiaceae, Orchidaceae, Scrophulariaceae, and Poaceae. The most important herbal species with PAs originate from the families Asteraceae (*Tussilago farfara*, *Petasites* sp., *Senecio* sp., *Adenostyles alliariae*, *Eupatorium* sp.), Boraginaceae (*Symphytum* sp., *Borago officnalis*, *Anchusa* *officinalis*, *Cynoglosum* *officinale*, *Echium* sp., *Heliotropium* sp., *Lithospermum* sp.) and Fabaceae (*Crotalaria* sp.) [6].

The genus *Rindera* Pall. belongs to Boraginaceae tribe *Cynoglosseae* DC. and includes about 25 species mostly distributed in central and western Asia [11]. *Rindera umbellata* is a biennial to perennial plant, found growing at sandy places in the Danube countries. In Serbia *R. umbellata* is narrowly distributed in Deliblatska peščara and Ramska peščara and it is only species of the genus which occurs in Serbia. The samples examined in this work were collected in Deliblatska peščara. The genus *Rindera* is known to be a rich source of PAs [12]. In this study, the PAs from the endemic plant *R. umbellata* were isolated and their structures were elucidated. 

The effect of lindelofine-*N*-oxide (**7**) on tubulin polymerization was determined. Tubulin is a globular protein crucial for cellular replication. It is heterodimer consisting of two closely related 55-kDa polypeptides called α-tubulin and β-tubulin, which polymerize to form hollow cylinders called microtubules. Microtubules exhibit differential dynamic behaviors during different phases of the cell cycle. Inhibition of the microtubule assembly dynamics causes cell cycle arrest leading to apoptosis, qualifying them as important drug targets for treating cancer [13,14]. Many anticancer natural products act by inhibiting or promoting the assembly of tubulin to microtubules [15]. To the best of our knowledge PAs have not been studied in this context so far.

The family Boraginaceae is one of the best known sources of fatty acids [16,17]. Fatty acids, which are widely occurred natural products [18,19,20] and have chemotaxonomic significance in Boraginaceae [17], were also identified in the seeds of this plant material.

## 2. Results and Discussion

**Figure 1:**
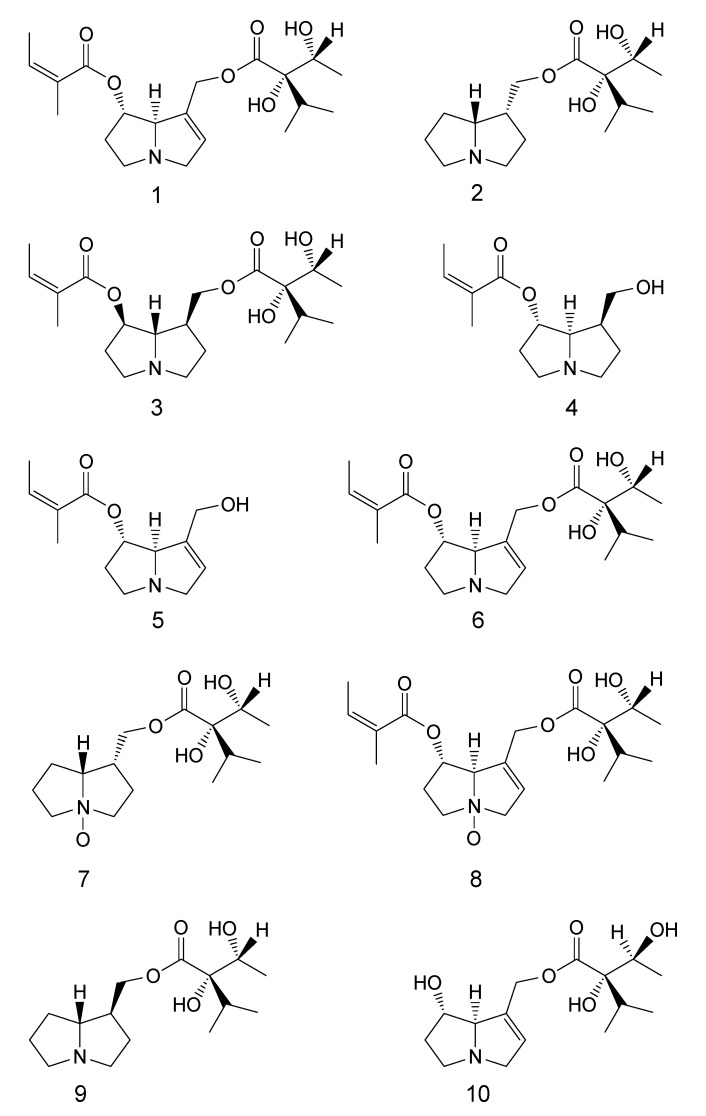
Structures of compounds **1**–**10**.

The isolation procedure for PAs from crude extracts of *R. umbellata* yielded ten alkaloids 1–10 (Figure 1, Table 1). Alkaloids **1**–**9** were isolated from the aerial parts of this plant. The extract of the roots gave PAs **7** and **8**. From the seeds of *R. umellata* PAs **6**–**10** were isolated. Three plant samples collected in different years were examined and yields of PAs depending on the plant’s parts and time of harvest are given in Table 2.

**Table 1:** IUPAC and common names of isolated PAs.

Compound number	IUPAC name	Common name
**1**	(*Z*)-((1*S*,7a*R*)-7-(((2*S*,3*R*)-2,3-dihydroxy-2-isopropylbutanoyloxy)methyl)-2,3,5,7a-tetrahydro-1*H*-pyrrolizin-1-yl)2-methylbut-2-enoate	7-Angeloyl-9-(+)-trachelanthylheliotridine
**2**	(2*S*,3*R*)-((1*R*,7a*R*)-hexahydro-1*H*-pyrrolizin-1-yl)methyl2,3-dihydroxy-2-isopropylbutanoate	Lindelofine
**3**	(*Z*)-((1*R*,7*S*,7a*S*)-7-(((2*S*,3*R*)-2,3-dihydroxy-2-isopropylbutanoyloxy)methyl)hexahydro-1*H*-pyrrolizin-1-yl) 2-methylbut-2-enoate	Punctanecine
**4**	(*Z*)-((1*S*,7*S*,7a*R*)-7-(hydroxymethyl)hexahydro-1*H*-pyrrolizin-1-yl) 2-methylbut-2-enoate	7-Angeloyl heliotridane
**5**	(*Z*)-((1*S*,7a*R*)-7-(hydroxymethyl)-2,3,5,7a-tetrahydro-1*H*-pyrrolizin-1-yl) 2-methylbut-2-enoate	7-Angeloyl heliotridine
**6**	(*Z*)-((1*S*,7a*R*)-7-(((*R*)-2,3-dihydroxy-2-((*R*)-1-hydroxyethyl)-3-methylbutanoyloxy)methyl)-2,3,5,7a-tetrahydro-1*H*-pyrrolizin-1-yl) 2-methylbut-2-enoate	Heliosupine
**7**	(2*S*,3*R*)-((1*R*,7a*R*)-hexahydro-1*H*-pyrrolizin-1-yl)methyl2,3-dihydroxy-2-isopropylbutanoate-*N*-oxide	Lindelofine- *N*-oxide
**8**	(*Z*)-((1*S*,7a*R*)-7-(((*R*)-2,3-dihydroxy-2-((*R*)-1-hydroxyethyl)-3-methylbutanoyloxy)methyl)-2,3,5,7a-tetrahydro-1*H*-pyrrolizin-1-yl) 2-methylbut-2-enoate-*N*-oxide	Heliosupine- *N*-oxide
**9**	(2*S*,3*R*)-((1*S*,7a*R*)-hexahydro-1*H*-pyrrolizin-1-yl)methyl 2,3-dihydroxy-2-isopropylbutanoate	9-(+)-Trachelanthyl-laburnine
**10**	(2*S*,3*R*)-((1S,7a*R*)-1-hydroxy-2,3,5,7a-tetrahydro-1*H*-pyrrolizin-7-yl)methyl 2,3-dihydroxy-2-isopropylbutanoate	Echinatine

**Table 2:** Quantities of PAs isolated from different parts of *Rindera* *umbellata*, in relation to time of harvest (mg/kg).

PA	Jun 2007 dry aerial parts	May 2008 dry aerial parts	May 2008 dry roots	July 2009 dry seeds
**1**		37.2		
**2**	233.3	141.6		66.9
**3**	7.1			
**4**		30.6		
**5**	5.7			
**6**	143.8	110		233.8
**7**	2051.0		113.2	94.4
**8**	238.6	270.2	130.3	62.5
**9**	91.9			
**10**				63.8

The structural assignments of alkaloids were based on comparison of their spectral data with those in literature [21,22].

In general, according to their necine bases, PAs could be classified into four types: heliotridine-type, platynecine-type, otonecine-type and retronecine-type. Retronecine and heliotridine are enantiomers at the C7 position, and besides otonecine-type PAs, have received the most attention because of their abundance and potent toxicities [6].

A rough estimation of PAs distribution within families Asteraceae and Boraginaceae indicated otonecine-type and heliotridine-type compounds as the main PAs, respectively. Retronecine-type PAs could be found in Fabaceae species, and also in Asteraceae and Boraginaceae species [6,12,23]. 

Most of isolated PAs from *R. umbellata* belong to the heliotridine-type. Correspondingly, heliotridine-type PAs were dominant in previously examined *Rindera* species: *R. austroechinata,* *R. baldschuanica*, *R. cyclodonta*, *R. echanata* and *R. oblongifolia* [12]. Echinatine was present in all of them, including *R. umbellata*, and it could be considered as a characteristic of the genus, although not enough *Rindera* species have been examined yet in order to conclude about PAs as chemical markers for distinguishing species within the genus.

Fatty acids of only few species of the genus *Rindera* have been previously analyzed and a new C_10_ unsaturated fatty acid (obtusilic acid) was found in the nuts of *R. obtusiloda* [24]. γ-Linolenic acid was identified in the seed oils of *Rindera lanata* and *R. umbellata* within a search of 33 species of the family Boraginaceae for a preferred source of this acid [25]. More detailed study of 45 species of the family confirmed that it is one of the best known sources of γ-linolenic acid, which is actually unusual in plants, but much appreciated because of its nutritional and medical benefits. Whereas in other families of angiosperms this acid is exclusively present in one or few genera, it has been found in most of the species of Boraginaceae evaluated to date, being absent or at very low concentrations only in the tribes Cordioideae, Ehretioideae and Heliotropioideae. Besides the presence of γ-linolenic acid, the concentration of linoleic, α- and γ-linolenic acid, stearidonic acid and erucic acid are of special chemotaxonomic importance within this family and follows some general rules at the tribal level. Regarding the tribe *Cynoglosseae* DC., the maximum concentrations of long-chain mono-unsaturated fatty acids were present in the comparison with other tribes of the family [17].

From the seeds of *R. umbellata* eleven fatty acids were isolated in the form of triglycerides. Fatty acids isolated from seeds were analysed after transesterification as methyl esters using GC-MS/FID. Palmitic (3.25%), linolenic (1.99%), linolic (16.60%), oleic (62.65%), stearic (1.40%), eicosenic (3.80%), eicosanic (trace), erucic (4.25%), docosanic (trace), nervonic (trace) and tetracosanic (trace) acids were identified by MS and their content was determined by FID. Due to the limited data about fatty acids in the genus *Rindera*, these data could not be appropriately interpreted within the genus, but it corresponds well to known facts about the fatty acids in the tribe *Cynoglosseae* DC. Although the high content of oleic acid was noticed in other analyzed plants of the tribe (24.0%–47.3%), *R. umbelata* differs due to the extremely high content of this monounsaturated acid (62.65%). 

The effect of lindelofine-*N*-oxide on tubulin polymerization was tested. Different concentrations of lindelofine-*N*-oxide (1, 10, 50 and 100 μM were incubated with tubulin solution and microtubule assembly was examined. The IC_50_ value for lindelofine-*N*-oxide was 91 μM, while the IC_50_ value for paclitaxel was 2.4 μM. Lindelofine-*N*-oxide thus exhibited a moderate effect on tubulin polymerization in comparison with paclitaxel. To the best of our knowledge effects of PAs on tubulin polymerization have not been previously studied. Despite the modest activity of lindelofine-*N*-oxide in this test, other PAs should be tested to verify their influence on tubulin polymerization and acting on this way as anticancer substances.

## 3. Experimental Section

### 3.1. General

The IR spectra were measured in the form of KBr pellets on a Perkin-Elmer FT-IR spectrometer 1725X. NMR spectra were recorded on a Varian Gemini 2000 (200 MHz for ^1^H) and a Bruker Avance III 500 (500 MHz for ^1^H) spectrometer with CDCl_3_ and CD_3_OD as solvents and TMS as reference. The mass spectra were obtained on a Finnigan MAT 8230, BE DCI (150 eV, isobutane).

GC-MS analyses were performed on an Agilent 7890A GC system equipped with a 5975C inert XL EI/CI MSD and a FID detector connected by capillary flow technology through a 2-way splitter with make-up gas. An HP-5 MS capillary column (Agilent Technologies, Santa Clara, CA, USA, 25 mm i.d., 30 m length, 0.25 μm film thickness) was used. 

Silica gel, 0.008 mm (Merck, Darmstadt, Germany), was used for preparative column chromatography (CC) and silica gel F-254 (Merck, Darmstadt, Germany) for analytical and preparative thin layer chromatography (TLC). The solvents were purified by distillation before use.

### 3.2. Plant Material

The plant material was collected during its flowering and fructification period in Deliblatska peščara, Serbia (June 2007, May 2008 and July 2009.). All tree harvests were performed at Latitude N 44°57'58'' and Longitude E 21°1'48''. Voucher specimens are deposited in the Herbarium of the Faculty of Biology, University of Belgrade.

### 3.3. Extraction and Isolation of PAs

The *R. umbellata* plant materials were air-dried at room temperature for 15 days.

#### 3.3.1. Harvest I (June 2007)

The dried and powdered aerial parts (210 g) were extracted with methanol (1.0 L) for 5 days at room temperature using an ultrasonic bath. After filtration and solvent removal, the residue (22 g) was dissolved in 1 M sulfuric acid and the pH adjusted to pH~2. The mixture was extracted with CH_2_Cl_2_ (2 × 70 mL). The aqueous layer was then made alkaline (pH~9.0) with NH_4_OH, and extracted with CH_2_Cl_2_ (3 × 150 mL). As the aqueous layer showed a positive Dragendorff test, it was extracted with *n*-butanol (2 × 50 mL). Both organic layers were dried with anhydrous magnesium sulphate. After filtration and solvent removal under reduced pressure, residues were purified by silica gel CC and prep. TLC to yield pure alkaloids. The first extract (16 g, from CH_2_Cl_2_,) was purified by column chromatography [silica gel, CH_2_Cl_2_-methanol-NH_4_OH (9:1:0.1)] and the polarity was gradually increased. One fraction was a mixture of two components (5 mg). After purification of the mixture by prep. TLC (CH_2_Cl_2_-methanol 9:1) **3** (1.5 mg) and **5** (1.2 mg) were isolated as a pure compounds. The results are shown in Table 2. The second extract (2 g, from *n*-BuOH) was purified by column chromatography (*n-*BuOH/MeOH/H_2_O/NH_3_ 70:15:10:5 v/v). The yield of isolated compound **7** was 430.7 mg (Table 2). The spots were detected under UV_254_, by Dragendorff reagent or by spraying with 50% H_2_SO_4_.

#### 3.3.2. Harvest II (May 2008)

The dried and powdered aerial parts (500 g) were extracted with methanol (2.4 L) for 5 days at room temperature using an ultrasonic bath. After filtration and solvent removal, the residue (38 g) was dissolved in 1 M sulfuric acid and the pH adjusted to pH~2. The mixture was extracted with CH_2_Cl_2_ (2 × 100 mL). The aqueous layer was then made alkaline (pH~9.0) with NH_4_OH, and extracted with CH_2_Cl_2_ (3 × 200 mL). The aqueous layer showed a negative Dragendorff test. The organic layer was dried with anhydrous magnesium sulphate. After filtration and solvent removal under reduced pressure the residue (17 g) was purified by column chromatography [silica gel, CH_2_Cl_2_-methanol-NH_4_OH (9:1:0.1)]. The yields of isolated compounds are shown in Table 2.

The dried and powdered roots (310 g) were extracted with methanol (1.0 L) for 5 days at room temperature using an ultrasonic bath. After filtration and solvent removal, the residue (10 g) was dissolved in 1 M sulfuric acid and the pH adjusted to pH~2. The mixture was extracted with CH_2_Cl_2_ (2 × 50 mL). The aqueous layer was then made alkaline (pH~9.0) with NH_4_OH, extracted with CH_2_Cl_2_ (3 × 50 mL). As the aqueous layer showed a positive Dragendorff test, it was extracted with *n*-butanol (2 × 50 mL). Both organic layers were dried with anhydrous magnesium sulphate. After filtration and solvent removal under reduced pressure residues were purified by silica gel CC to yield pure alkaloids. The first extract (3 g, from CH_2_Cl_2_) was purified by column chromatography [silica gel, CH_2_Cl_2_-methanol-NH_4_OH (9:1:0.1)]. Only compound 8 (40.4 mg) was isolated from the dichloromethane extract. The second extract (3.7 g, from *n*-BuOH) was purified by column chromatography (*n-*BuOH-MeOH-H_2_O-NH_3_ 70:15:10:5, v/v). Only compound **7** (35.1 mg) was isolated from the butanolic extract (Table 2). 

#### 3.3.3. Harvest III (July 2009)

The dried and powdered seeds (160 g) were extracted with methanol (0.6 L) for 5 days at room temperature using an ultrasonic bath. After filtration and solvent removal, the residue (22 g) was dissolved in 1 M sulfuric acid and the pH adjusted to pH~2. The mixture was extracted with CH_2_Cl_2_ (2 × 50 mL). The aqueous layer was then made alkaline (pH~9.0) with NH_4_OH, extracted with CH_2_Cl_2_ (3 × 50 mL). As the aqueous layer showed a positive Dragendorff test, it was extracted with *n*-butanol (2 × 30 mL). Both organic layers were dried with anhydrous magnesium sulphate. After filtration and solvent removal under reduced pressure residues were purified by silica gel CC to yield pure alkaloids. The first extract (343 mg, from CH_2_Cl_2_) was purified by column chromatography [silica gel, CH_2_Cl_2_-methanol-NH_4_OH (9:1:0.1)]. The quantities of isolated compounds are shown in Table 2. The butanolic extract (1.0 g) was purified by column chromatography (*n-*BuOH-MeOH-H_2_O-NH_3_ 70:15:10:5, v/v). Only compound **7** (15.1 mg) was isolated (Table 2) from the butanolic extract. 

### 3.4. Compound Characterization

#### 3.4.1. Compound **1**

Colorless oil; CIMS [M+H]^+^ *m/z* 382; ^1^H-NMR (200 MHz, CDCl_3_) δ: 6.2 (1H, qq, *J_1_* = 6.9 Hz, *J_2_* = 1.4 Hz, H-3''), 5.85 (1H, br s, H-2), 5.17 (1H, br s, H-7), 4.95 (2H, br s, H-9), 4.09 (1H, q, *J* = 6.4 Hz, H-3'), 4.06 (1H, m, H-8), 3.96 (1H, br d, H-3b), 3.36 (1H, m, H-3a), 3.20 (1H, m, H-5b), 2.86 (1H, m, H-5a), 2.08 (1H, m, *J* = 6.9 Hz, H-5'), 1.98 (3H, m, *J_1_* = 6.9 Hz, *J_2_* = 1.4 Hz, H-4''), 1.92 (2H, m, H-6), 1.87 (3H, m, *J* = 1.4 Hz, H-5''), 1.27 (3H, d, *J* = 6.4 Hz, H-4'), 0.94 (3H, d, *J* = 6.6 Hz, H-6'), 0.93 (3H, d, *J* = 6.9 Hz, H-7'); ^13^C-NMR (50 MHz, CDCl_3_) δ: 175.2 (s, C-1'), 168.1 (s, C-1''), 138.9 (d, C-3''), 134.5 (s, C-1), 129.3 (d, C-2), 127.6 (s, 2''), 82.9 (s, 2'), 79.2 (d, C-8), 76.9 (d, C-7), 69.3 (d, C-3'), 62.2 (t, C-3), 62.2 (t, C-9), 54.2 (t, C-5), 32.8 (d, C-5’), 30.2 (t, C-6), 20.4 (q, C-5''), 17.3 (q, C-4'), 17.1 (q, C-6'), 16.7 (q, C-7'), 15.8 (q, C-4'').

#### 3.4.2. Compound **2**

White crystals; CIMS [M+H]^+^ *m/z* 286; ^1^H-NMR (200 MHz, CDCl_3_) δ: 4.30 (2H, br d, *J* = 7.2 Hz, H-9), 4.09 (1H, q, *J* = 6.4 Hz, H-3'), 3.66 (1H, m, H-8), 3.43 (1H, m, H-3a), 2.91 (1H, m, H-5a), 2.65 (1H, m, H-5b), 2.50 (1H, m, H-3b), 2.00 (1H, m, H-1), 1.96 (1H, m, H-5'), 1.70 (2H, m, H-2), 1.60 (2H, m, H-6), 1.53 (2H, m, H-7), 1.21 (3H, d, *J* = 6.2 Hz, H-4'), 0.97 (3H, d, *J* = 7.2 Hz, H-7'), 0.92 (3H, d, *J* = 6.6 Hz, H-6'); ^13^C-NMR (50 MHz ,CDCl_3_) δ: 174.8 (s, C-1'), 83.0 (s, C-2'), 69.1 (d, C-3'), 67.0 (d, C-8), 64.4 (t, C-9), 55.7 (t, C-5), 53.6 (t, C-3), 39.8 (d, C-1), 32.9 (d, C-5'), 26.2 (t, C-2), 25.8 (t, C-7), 25.7 (t, C-6), 16.9 (q, C-7'), 16.8 (q, C-6'), 16.4 (q, C-4').

#### 3.4.3. Compound **3**

Colorless oil; CIMS [M+H]^+^ *m/z* 384; ^1^H-NMR (200 MHz, CDCl_3_) δ: 6.10 (1H, qq, *J_1_* = 7.9 Hz, *J_2_* = 1.4 Hz, H-3''), 5.10 (1H, dd , H-7), 4.38 (2H, dd, H-9), 4.04 (1H, m, H-3'), 3.60 (1H, m, H-8), 3.22 (1H, m, H-3b), 2.66 (1H, m, H-3a), 3.02 (1H, m, H-5b), 2.75 (1H, m, H-5a), 2.14 (1H, m, *J* = 6.6 Hz, H-5'), 2.02 (2H, m, H-2), 2.00 (1H, m, H-1), 2.00 (1H, m, H-6b), 1.97 (3H, m, *J_1_* = 7.4 Hz, *J_2_* = 1.4 Hz, H-4''), 1.94 (1H, m, H-6a), 1.87 (3H, m, *J* = 1.4 Hz, H-5''), 1.21 (3H, d, *J* = 6.6 Hz, H-4'), 0.93 (3H, d, *J* = 6.8 Hz, H-6'), 0.92 (3H, d, *J* = 6.9 Hz, H-7').

#### 3.4.4. Compound **4**

Colorless oil; CIMS [M+H]^+^ *m/z* 240; ^1^H-NMR (200 MHz, CDCl_3_) δ: 6.13 (1H, qq, *J_1_* = 7.3 Hz, *J_2_* = 1.4 Hz, H-3''), 5.19 (1H, m, H-7), 3.79 (2H, d, *J* = 6.9 Hz, H-9), 3.50 (1H, m, H-8), 3.24 (1H, m, H-5b), 3.06 (1H, m, H-3b), 2.83 (1H, m, H-5a), 2.63 (1H, m, H-3a), 2.63 (1H, m, H-1), 2.00 (2H, m, H-6), 1.99 (3H, m, *J_1_* = 7.2 Hz, *J_2_* = 1.4 Hz, H-4''), 1.86 (3H, m, H-5''), 1.40 (2H, m, H-2); ^13^C-NMR (50 MHz, CDCl_3_) δ: 168.5 (s, C-1''), 139.4 (d, C-3''), 127.4 (s, 2''), 74.8 (d, C-8), 72.6 (d, C-7), 63.2 (t, C-9), 54.3 (t, C-3), 53.8 (t, C-5), 44.5 (d, C-1), 32.7 (t, C-6), 28.8 (t, C-2), 20.4 (q, C-5''), 15.9 (q, C-4'').

#### 3.4.5. Compound **5**

Colorless oil; CIMS [M+H]^+^ *m/z* 238; ^1^H-NMR (200 MHz, CDCl_3_) δ: 6.13 (1H, qq, *J_1_* = 6.5 Hz, *J_2_* = 1.4 Hz, H-3''), 5.62 (1H, br s, H-2), 5.12 (1H, m, C-7), 4.36 (2H, br s, H-9), 4.06, (1H, m, H-8), 3.90 (1H, m, H-3b), 3.32 (1H, m, H-3a), 3.16 (1H, m, H-5b), 2.85 (1H, m, H-5a), 2.00 (3H, m, *J_1_* = 7.4 Hz, *J_2_* = 1.4 Hz, H-4''), 1.91 (2H, m, H-6), 1.87 (3H, m, *J* = 1.4 Hz, H-5'').

#### 3.4.6. Compound **6**

Yellow oil; EIMS [M]^+^ *m/z* 397; ^1^H-NMR (500 MHz, CDCl_3_) δ: 6.16 (1H, qq, *J_1_* = 7.2 Hz, *J_2_* = 1.5 Hz, H-3''), 5.92 (1H, br s, H-2), 5.24 (1H, m, H-7), 5.04 (1H, br d, *J* = 13 Hz, H-9a), 4.93 (1H, br d, *J* = 13 Hz, H-9b), 4.21 (1H, q, *J* = 6.5 Hz, H-3'), 4.41 (1H, m, H-8), 4.16 (1H, m, H-3a), 3.47 (1H, m, H-3b), 3.38 (1H, m, H-5a), 2.97 (1H, m, H-5b), 2.04 (2H, m, H-6), 1.98 (3H, dq, *J_1_* = 7.2 Hz, *J_2_* = 1.5 Hz, H-4''), 1.88 (3H, m, *J* = 1.5 Hz, H-5''), 1.30 (3H, s, H-7'), 1.28 (3H, d, *J* = 7.0 Hz, H-4'), 1.26 (3H, s, H-6'); ^13^C-NMR (125 MHz, CDCl_3_) δ: 174.3 (s, C-1'), 168.1 (s, C-1''), 139.8 (s, C-1), 139.7 (d, C-3''), 128.9 (d, C-2), 127.2 (s, 2''), 82.8 (s, 2'), 79.1 (d, C-8), 76.7 (d, C-7), 73.8 (s, C-5'), 69.8 (d, C-3'), 62.0 (t, C-9), 61.9 (t, C-3), 54.1 (t, C-5), 30.0 (t, C-6), 25.9 (q, C-6'), 24.8 (q, C-7'), 20.4 (q, C-5''), 18.5 (q, C-4'), 15.9 (q, C-4'').

#### 3.4.7. Compound **7**

White crystals; CIMS [M+H]^+^ *m/z* 302; ^1^H-NMR (600 MHz, CDCl_3_) δ: 4.28 (1H, dd, *J_1_* = 10.8 Hz, *J_2_* = 8.4 Hz, 9a), 4.14 (1H, m, *J_1_* = 11.4 Hz, *J_2_* = 6.6 Hz, 9b), 4.10 (1H, m, H-8), 4.08 (1H, q, *J* = 6.6 Hz, H-3'), 3.73 (1H, m, H-5b), 3.66 (1H, m, H-3a), 3.62 (1H, m, H-3b), 3.51 (1H, m, H-5b), 3.27 (1H, m, *J* = 8,4 Hz, H-1), 2.42 (1H, m, H-2a), 2.35 (1H, m, H-6a), 2.28 (1H, m, H-7a), 1.98 (1H, m, H-6b), 1.86 (1H, m, *J* = 6.6 Hz, H-5'), 1.75 (1H, m, H-2b), 1.69 (1H, m, H-7b), 1.18 (3H, d, *J* = 6.6 Hz, H-4'), 0.95 (3H, d, *J* = 7,2 Hz, H-7'), 0.90 (3H, d, *J* = 7,2 Hz, H-6'); ^13^C-NMR (150 MHz, CDCl_3_) δ: 175.1 (s, C-1'), 83.9 (d, C-8), 83.5 (s, C-2'), 69.8 (d, C-3'), 69.4 (t, C-5), 68.2 (t, C-3), 63.5 (t, C-9), 37.8 (d, C-1), 33.5 (d, C-5'), 26.6 (t, C-2), 25.1 (t, C-7), 22.8 (t, C-6), 17.6 (q, C-7'), 17.2 (q, C-6'), 16.9 (q, C-4').

#### 3.4.8. Compound **8**

Yellow oil; EIMS [M^+^–16] *m/z* 397; ^1^H-NMR (500 MHz, CDCl_3_) δ: 6.22 (1H, qq, *J_1_* = 7.2 Hz, *J_2_* = 1.5 Hz, H-3''), 6.03 (1H, br s, H-2), 5.21 (1H, br d, *J* = 13 Hz, H-9a), 5.09 (1H, br s, H-7), 4.86 (1H, br d, *J* = 13 Hz, H-9b), 4.73 (1H, s, H-8), 4.58 (1H, dd, *J* = 16 Hz, H-3b), 4.46 (1H, dd, *J* = 16 Hz, H-3a), 4.19 (1H, q, *J* = 6.5 Hz, H-3'), 3.94 (1H, m, H-5a), 3.84 (1H, m, H-5b), 2.52 (1H, m, H-6a), 2.26 (1H, m, H-6b), 2.03 (3H, dq, *J_1_* = 7.2 Hz, *J_2_* = 1.5 Hz, H-4''), 1.92 (3H, m, *J* = 1.5 Hz, H-5''), 1.31 (3H, s, H-7'), 1.27 (3H, d, *J* = 6.5 Hz, H-4'), 1.24 (3H, s, H-6'); ^13^C-NMR-(125 MHz, CDCl_3_) δ: 174.3 (s, C-1'), 167.4 (s, C-1''), 141.2 (d, C-3''), 132.7 (s, C-1), 126.5 (s, 2''), 123.8 (d, C-2), 94.7 (d, C-8), 84.2 (s, C-2'), 77.0 (t, C-3), 73.2 (s, C-5'), 73.1 (d, C-7), 69.6 (d, C-3'), 67.6 (t, C-5), 60.6 (t, C-9), 30.5 (t, C-6), 26.4 (q, C-6'), 24.7(q, C-7'), 20.3 (q, C-5''), 18.6 (q, C-4'), 16.0 (q, C-4'').

#### 3.4.9. Compound **9**

Yellow oil; CIMS [M+H]^+^ *m/z* 286; ^1^H-NMR (200 MHz, CDCl_3_) δ: 4.27 (2H, m, *J* = 7.2 Hz, H-9), 4.06 (1H, q, *J* = 6.4 Hz, H-3'), 3.66 (1H, m, H-8), 3.31 (1H, m, H-3a), 3.14 (1H, m, H-5a), 2.65 (1H, m, H-5b), 2.50 (1H, m, H-3b), 2.00 (1H, m, H-1), 1.99 (1H, m, *J* = 7,0 Hz, H-5'), 1.90 (2H, m, H-2), 1.80 (2H, m, H-6), 1.68 (2H, m, H-7), 1.21 (3H, d, *J* = 6.2 Hz, H-4'), 0.96 (3H, d, *J* = 7.2 Hz, H-7'), 0.92 (3H, d, *J* = 6.8 Hz, H-6'); ^13^C-NMR (50 MHz, CDCl_3_) δ: 174.8 (s, C-1'), 82.8 (s, C-2'), 68.8 (d, C-3'), 66.0 (d, C-8), 65.1 (t, C-9), 55.4 (t, C-5), 53.4 (t, C-3), 39.8 (d, C-1), 32.7 (d, C-5'), 26.6 (t, C-2), 25.9 (t, C-7), 25.7 (C-6), 16.8 (q, C-7'), 16.7 (q, C-6'), 16.6 (q, C-4').

#### 3.4.10. Compound **10**

Colorless oil; EIMS [M]^+^ *m/z* 299; ^1^H-NMR: (200 MHz, CDCl_3_) δ: 5.72 (1H, br s, H-2), 4.90 (2H, m, *J* = 13.8 Hz, H-9), 4.25 (1H, m, C-7), 4.19 (1H, m, H-8), 4.03 (1H, q, H-3a), 3.97 (1H, q, *J* = 6.6 Hz, H-3'), 3.44 (1H, m, H-3b), 3.38 (1H, m, H-5a), 2.72 (1H, m, H-5b), 2.18 (1H, m, *J* = 6.9 Hz, H-5'), 1.93 (2H, m, H-6), 1.30 (3H,d, *J* = 6.6 Hz, H-4'), 0.93 (3H, d, *J* = 6.9 Hz, H-6'), 0.87 (3H, d, *J* = 6.9 Hz, H-7'); ^13^C-NMR (50 MHz, CDCl_3_) δ: 174.1(s, C-1'), 135.7 (s, C-1), 125.4 (d, C-2), 84.1 (s, C-2'), 79.8 (d, C-8), 73.6 (d, C-7), 71.6 (d, C-3'), 61.7 (t, C-3), 61.4 (t, C-9), 54.3 (t, C-5), 33.4 (t, C-6), 32.2 (d, C-5'), 17.8 (q, C-7'), 17.1 (t, C-4'), 15.7 (q, C-6').

### 3.5. Extraction of Fatty Acids and GC-MS/FID Analyses

The dried and powdered seeds (10 g) were extracted with petroleum ether (0.4 L) for 2 days at room temperature using an ultrasonic bath. The solvent was removed under reduced pressure and the residue (450 mg) was dissolved in absolute methanol (20 mL) containing conc.H_2_SO_4_ (0.1 mL) and refluxed for two hours. After cooling the reaction mixture, the methanol was removed under reduced pressure and residue was dissolved in water, extracted with CH_2_Cl_2_ (2 × 30 mL), neutralised with saturated sodium bicarbonate solution (2 × 20 mL) and dried over anhydrous magnesium sulphate. After filtration, the solvent was removed and residue (254 mg) was analysed using GC-MS. Samples were injected in splitless mode. The injection volume was 1 μL, and the injector temperature was 250 °C. The carrier gas (He) flow rate was 1.1 mL/min, whereas the column temperature was programmed linearly in a range of 40–240 °C at a rate of 4 °C/min. The transfer line temperature was 280 °C. The FID detector temperature was 300 °C. EI mass spectra (70 eV) were acquired in the *m/z* range of 45–450, and the ion source temperature was 230 °C.

### 3.6. Tubulin Polymerization

The effect of lindelofine-*N*-oxide on tubulin polymerization was determined using a standard method [26], with some modifications [27]. Tubulin was isolated from a bovine brain as described previously [28]. Freshly prepared tubulin solution (2.2 mg mL^−1^) and MES (2-(*N*-morpholino) ethanesulfonic acid) buffer containing guanosine triphosphate (GTP) were kept on ice before the experiment. Stock solutions of paclitaxel and lindelofine-*N*-oxide were prepared in DMSO at concentration of 10 mM, and afterwards diluted with DMSO/H_2_0 (1:1 v/v) to a concentration of 1 mM. From this solution, the desired concentrations (in the range of 1–1,000 μM) were prepared in H_2_O. Solutions (40 μL) of various concentrations of either PTX (positive control) or lindelofine-*N*-oxide were added to a tubulin solution (460 μL) and incubated for 45 min at 37 °C. The mixture of MES buffer (40 μL) and tubulin (460 μL) was used as blank. After incubation, solutions were transferred to UV cuvettes and absorbance was measured at 350 nm continuously for 15 min on a GBC Cintra 40 UV-Visible spectrometer equipped with a Petrotest 25-0395 thermostatic circulator cooled to 4 °C. A percentage of tubulin polymerization was determined as difference in absorbance at *t* = 0 min (37 °C) and *t* = 15 min (4 °C), compared to corresponding difference for the blank. The effect of paclitaxel and lindelofine-*N*-oxide on polymerization of purified tubulin was expressed as concentration of each agent producing 50% tubulin polymerization (IC_50_).

## 4. Conclusions

To the best of our knowledge this is the first report about phytochemical investigation of *Rindera* *umbellata.* Ten PAs were isolated and identified from the aerial parts, roots and seeds of this endemic plant. Eleven fatty acids were also identified from the seeds. The results could be of chemotaxonomic importance, because *R. umbelatte* was examined for the first time. Lindelofine-*N*-oxide exhibited a moderate effect on tubulin polymerization. 
